# Exploring the inhibitory potential of the antiarrhythmic drug amiodarone against *Clostridioides difficile* toxins TcdA and TcdB

**DOI:** 10.1080/19490976.2023.2256695

**Published:** 2023-09-25

**Authors:** Judith Schumacher, Astrid Nienhaus, Sebastian Heber, Jauheni Matylitsky, Esteban Chaves-Olarte, César Rodríguez, Holger Barth, Panagiotis Papatheodorou

**Affiliations:** a Institute of Experimental and Clinical Pharmacology, Toxicology and Pharmacology of Natural Products, Ulm University Medical Center, Ulm, Germany; b Centro de Investigación en Enfermedades Tropicales and Facultad de Microbiología, Universidad de Costa Rica, San José, Costa Rica

**Keywords:** *Clostridioides difficile* infection, toxin inhibitor, membrane cholesterol, drug repurposing, drug repositioning

## Abstract

The intestinal pathogen *Clostridioides difficile* is the leading cause of antibiotic-associated diarrhea and pseudomembranous colitis in humans. The symptoms of *C. difficile*-associated diseases (CDADs) are directly associated with the pathogen’s toxins TcdA and TcdB, which enter host cells and inactivate Rho and/or Ras GTPases by glucosylation. Membrane cholesterol is crucial during the intoxication process of TcdA and TcdB, and likely involved during pore formation of both toxins in endosomal membranes, a key step after cellular uptake for the translocation of the glucosyltransferase domain of both toxins from endosomes into the host cell cytosol. The licensed drug amiodarone, a multichannel blocker commonly used in the treatment of cardiac dysrhythmias, is also capable of inhibiting endosomal acidification and, as shown recently, cholesterol biosynthesis. Thus, we were keen to investigate *in* *vitro* with cultured cells and human intestinal organoids, whether amiodarone preincubation protects from TcdA and/or TcdB intoxication. Amiodarone conferred protection against both toxins independently and in combination as well as against toxin variants from the clinically relevant, epidemic *C. difficile* strain NAP1/027. Further mechanistic studies suggested that amiodarone’s mode-of-inhibition involves also interference with the translocation pore of both toxins. Our study opens the possibility of repurposing the licensed drug amiodarone as a novel pan-variant antitoxin therapeutic in the context of CDADs.

## Introduction

Antibiotics-associated diarrhea and pseudomembranous colitis are two dreaded diseases that are mainly caused after infection with the (nosocomial) human gut pathogen *Clostridioides difficile*. Clinical symptoms after *C. difficile* infections (CDIs) are strictly related to the action of two independent but highly similar protein toxins of this pathogen, namely TcdA (toxin A) and TcdB (toxin B), on target cells in the human gut.^1–3^

Both toxins, TcdA and TcdB, are prototype members of the family of clostridial glucosylating toxins (CGTs), which inactive Rho and/or Ras proteins in the host cell cytosol by covalent attachment of a glucose moiety (mono-O-glucosylation). Because Rho family members are, inter alia, master regulators of the actin cytoskeleton, their inactivation by TcdA/TcdB leads to the destruction of the actin meshwork and hence to cell rounding as a typical morphological feature of intoxicated cultured cells growing in monolayers.^4–6^

TcdA and TcdB are single-chain, multidomain toxins that are taken up into host cells via receptor-mediated endocytosis, depending either on clathrin (TcdB) and/or PACSIN2 (TcdA).^7–9^ Several TcdA- and TcdB-specific host entry receptors have been identified so far, which interact either with a C-terminally located domain called CROP (combined repetitive oligopeptides) or with additional receptor-binding modules preceding the CROP domain.^10,11^ Upon acidification of the endocytic vesicles by vesicular adenosine triphosphatases (V-ATPases), the middle part of the toxins, the so-called translocation domain (TD), alters its structure and inserts into the endosomal membrane.^12,13^ Thus, a membrane pore is formed by the TD which enables the translocation of the N-terminally located glucosyltransferase domain (GTD) and the adjacent cysteine protease domain (CPD) of the toxins into the cytosol.^14^ Eventually, the cytosolic molecule inositol hexakisphosphate (InsP6) binds and activates the CPD of the toxins for autocatalytic cleavage and release of the GTD into the cytosol.^15,16^

Successive studies have shown that cholesterol plays a key role in the ability of TcdA and TcdB to insert and form translocation pores in endosomal membranes. For instance, decreasing the cellular cholesterol levels in cultured human cells with substances that interfere with cholesterol biosynthesis and/or intracellular transport, such as simvastatin, 25-hydroxycholesterol, PF-429242, and U18666A, protected cells from intoxication with TcdA and TcdB, respectively. In particular, these compounds conferred protection to cells also against TcdA and TcdB variants isolated from the epidemic *C. difficile* strain NAP1/027.^17–19^

Given the important role of membrane cholesterol during the intoxication process of TcdA and TcdB, compounds that interfere with cholesterol biosynthesis might represent putative pharmacological inhibitors of both toxins. In this context, we became aware of recent studies revealing that the licensed antiarrhythmic drug amiodarone, which is a multichannel blocker commonly used in the treatment of cardiac dysrhythmias,^20^ inhibited cholesterol biosynthesis in cultured human cells.^21,22^ As shown in two independent studies, amiodarone was capable of directly inhibiting the 24-dehydrocholesterol reductase (DHCR24) enzymatic activity in microsomal membranes and resulted in accumulation of the cholesterol precursor molecule desmosterol in cultured human cells.^21,22^ Just recently, amiodarone was found to exert an antiviral effect by the depletion of membrane cholesterol.^23^

Prompted by the rather unexpected finding of the studies mentioned above about the pharmacological action of amiodarone on cholesterol biosynthesis, we investigated whether cultured mammalian and human cells as well as ‘miniguts’ (human intestinal organoids; HIOs) pretreated with this medicinal compound are less prone to intoxication by TcdB. In line with our working hypothesis, amiodarone exhibited a protective effect against TcdB in all tested cell lines and in ‘miniguts’. Moreover, it conferred protection to cultured cells also against TcdA, the medically pertinent combination of TcdA and TcdB, as well as against TcdA and TcdB from the clinically relevant, epidemic *C. difficile* strain NAP1/027. We then sought out to understand the exact mechanism behind amiodarone’s inhibitory activity against TcdA and TcdB. Surprisingly, our data indicates that amiodarone’s mode-of-inhibition likely involves interference with pore formation and translocation of both toxins, respectively.

Since amiodarone represents an already approved licensed drug, our study points toward the possibility of repurposing this drug as a novel pan-variant antitoxin therapeutic in the context of *C. difficile*-associated diseases (CDADs).

## Results

### Vero cells preincubated for 24 h with amiodarone are protected from TcdB

To study whether amiodarone is worth to be considered as a potential pharmacological inhibitor of TcdA and TcdB, we used from the start natively purified TcdB from the historical *C. difficile* strain VPI 10463 as representative for both glucosylating toxins of *C. difficile* and African green monkey kidney (Vero) cells as established *in vitro* model system for intoxication experiments.

**Figure 1.**
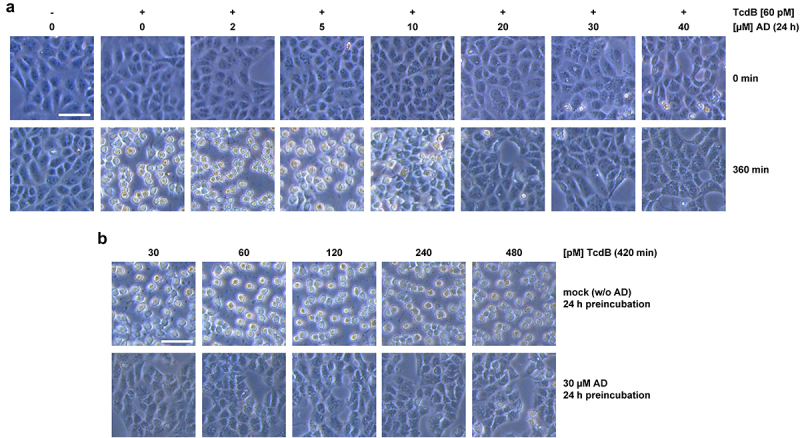
Effect of 24 h amiodarone preincubation on the intoxication of Vero cells by TcdB. (a) Vero cells were preincubated for 24 h with increasing concentrations of amiodarone (AD) as indicated, prior to addition of 60 pM TcdB (+ TcdB). Control cells were left without toxin treatment (− TcdB). Shown are microscopic images from time points 0 (upper row) and 360 min (lower row) after TcdB addition. (b) microscopic images from Vero cells preincubated for 24 h either without amiodarone (mock; w/o AD; upper row) or with 30 µM amiodarone (30 µM AD; lower row), followed by the intoxication of the cells for 420 min with increasing concentrations of TcdB as indicated. Scale bar shown in (a) and (b) represents 100 µm.

Based on the recent findings that amiodarone is capable of inhibiting cholesterol biosynthesis in cells,^21,22^ we assumed that a rather long-term preincubation period of cells with amiodarone might be necessary, to decrease the membrane cholesterol level efficiently. We therefore preincubated Vero cells for 24 h with increasing concentrations of amiodarone, prior to intoxication of the cells with 60 pM TcdB and microscopic analysis of TcdB-induced cell rounding. This cytopathic effect is a typical, specific, and well-established hallmark of intoxication of cultured cells with TcdB and TcdA, respectively. In cells without amiodarone, TcdB-induced cell rounding was obvious and complete after 360 min (Figure 1a). Interestingly, inhibition of TcdB-induced cell rounding became evident in cells preincubated with at least 20 µM amiodarone (Figure 1a).

For obtaining a robust inhibitory effect against TcdB in Vero cells, we decided to use 30 µM amiodarone for further testing of the inhibitory potential of the compound against increasing TcdB concentrations (up to 480 pM). As can be seen in exemplary microscopic images of Vero cells intoxicated for 420 min with TcdB, preincubation of the cells for 24 h with 30 µM amiodarone conferred almost complete protection against TcdB-induced cell rounding at all tested toxin concentrations (Figure 1b).

### One-hour preincubation of Vero cells with amiodarone is sufficient to protect cells from TcdB intoxication

**Figure 2.**
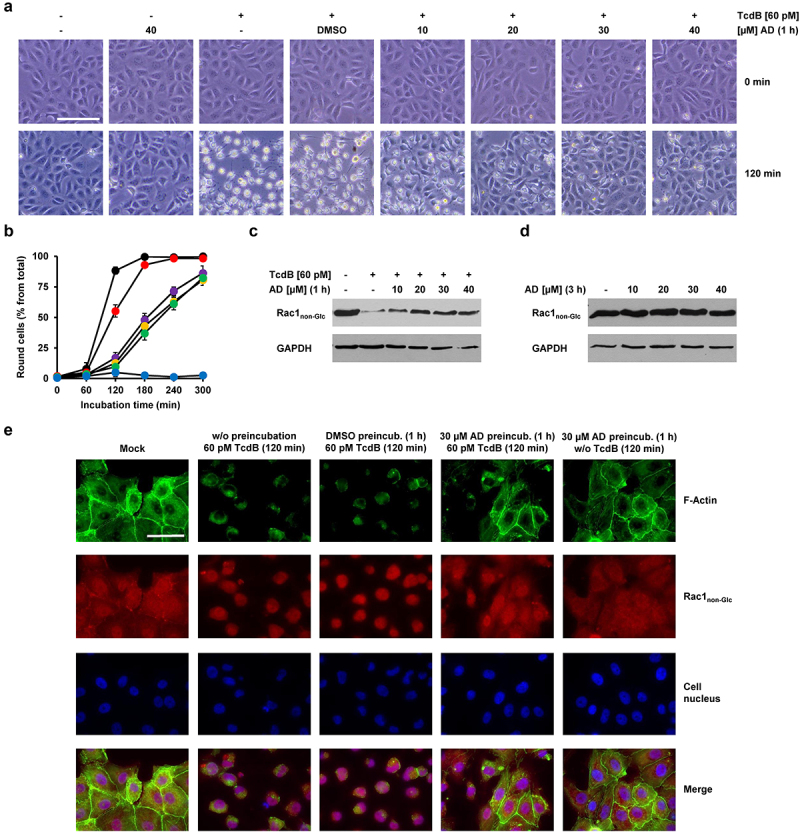
Effect of 1 h amiodarone preincubation on the intoxication of Vero cells by TcdB. (a) Vero cells were preincubated for 1 h with increasing concentrations of amiodarone (AD) as indicated, without amiodarone (− AD) or only with solvent (DMSO), prior to addition of 60 pM TcdB (+ TcdB). Control cells were left without toxin treatment (− TcdB). Shown are representative microscopic images from time points 0 (upper row) and 120 min (lower row) after TcdB addition. (b) quantification of cell rounding over time in mock- (black filled circles) and 1 h amiodarone-preincubated (10 µM, red filled circles; 20 µM purple filled circles; 30 µM, yellow filled circles; 40 µM green filled circles) Vero cells intoxicated with 60 pM TcdB or without amiodarone preincubation and toxin addition (blue filled circles). Shown are the mean percentage values of round cells (in % from total cells) as calculated from three parallel experiments in independent wells. Error bars represent ±SD. (c) effect of amiodarone on TcdB-induced Rac1 glucosylation in Vero cells. Cells were preincubated for 1 h with increasing concentrations of amiodarone (AD) as indicated or without amiodarone (− AD), followed by intoxication with 60 pM TcdB for 120 min (+ TcdB) or without TcdB (− TcdB) and generation of whole-cell lysates. Representative immunoblots against non-glucosylated Rac1 (Rac1_non-Glc_) and GAPDH (loading control) are shown. In a control sample cells were left without amiodarone (− AD) preincubation and toxin addition (− TcdB). (d) immunoblots as shown in (c) but with whole-cell lysates obtained from Vero cells were preincubated for 3 h only with the indicated concentrations of amiodarone (AD). (e) Vero cells either preincubated with 30 µM amiodarone (30 µM AD preincub.) for 1 h, with DMSO solvent only (DMSO preincub.) or without preincubation (w/o preincubation) were further incubated for 120 min with 60 pM TcdB or without TcdB (w/o TcdB). Control cells (mock) were neither preincubated with amiodarone or DMSO solvent nor with TcdB. Fluorescence images obtained with a 60× oil objective, represent Hoechst 33342-staining of cell nuclei (colored in blue), phalloidin-FITC-staining of actin filaments (F-actin; colored in green), immunostaining (Alexa Fluor® 568) of non-glucosylated Rac1 (Rac1_non-Glc_; colored in red), and the overlay of the individual fluorescence channels (merge). Scale bar in (a) represents 100 µm and in (e) 50 µm.

Next, we tested whether a short-term preincubation of Vero cells with amiodarone is sufficient to protect cells from TcdB intoxication. It is highly unlikely that cholesterol levels in cell membranes are substantially decreasing within the first hours after pharmacological inhibition of cholesterol biosynthesis. Therefore, we preincubated Vero cells for only 1 h with increasing concentrations of amiodarone, prior to the intoxication of the cells for 120 min with 60 pM TcdB. To our surprise, preincubation of the cells for 1 h with 20 to 40 µM amiodarone was sufficient to inhibit TcdB-induced cell rounding (Figure 2a). Quantification of TcdB-induced cell rounding over time revealed a strong delay of intoxication in amiodarone-preincubated cells, with a maximal reduction in cell rounding of about 75% at time point 120 min in cells preincubated for 1 h with 20 to 40 µM amiodarone (Figure 2b).

To confirm these findings with a more specific biochemical readout of TcdB intoxication, Vero cells were preincubated for 1 h with increasing concentrations of amiodarone, prior to intoxication of the cells for 120 min with 60 pM TcdB. Then, the glucosylation status of the TcdB target substrate Rac1 was analyzed in whole-cell lysates by immunoblotting involving a Rac1-specific, glucosylation-sensitive antibody that does only recognize the non-glucosylated form of Rac1 (denoted as Rac1_non-Glc_ antibody). In this context, a decrease of Rac1_non-Glc_ signals in immunoblots is directly indicative of TcdB intoxication and hence cellular uptake of the toxin. As expected, the Rac1_non-Glc_ signal was strongly decreased in whole-cell lysates obtained from TcdB-intoxicated Vero cells without amiodarone preincubation. However, Rac1_non-Glc_ signals were increased in whole-cell lysates from amiodarone-preincubated cells (Figure 2c). Amiodarone itself (3 h incubation with the cells in total) had no influence on the glucosylation status and/or levels of Rac1 (Figure 2d).

In addition, we implemented fluorescence microscopy to confirm the inhibitory potential of amiodarone on the intoxication of Vero cells with TcdB. Cells preincubated for 1 h either with or without amiodarone (30 µM) (or with DMSO solvent only), were intoxicated for 120 min with 60 pM TcdB, followed by staining of F-actin with fluorescently labeled phalloidin (phalloidin-FITC) and Rac1_non-Glc_-immunostaining, respectively. Intoxication of Vero cells with TcdB led clearly to the collapse of the actin cytoskeleton, whereas in amiodarone-preincubated cells, F-actin structures and cytosolic Rac1 signals were preserved after TcdB intoxication (Figure 2e).

It can already be concluded from these findings that the cell protective effect of amiodarone against TcdB intoxication seems not to be solely associated with decreased membrane cholesterol levels due to the inhibition of cholesterol biosynthesis by amiodarone.

### TcdB intoxication is delayed also in HeLa and CaCo-2 cells preincubated for 1 h with amiodarone

Next, we were prompted to test whether amiodarone confers protection against TcdB also in human cells, such as the cervical carcinoma cell line HeLa and – for the toxins of a gut pathogen – the physiologically more relevant colon carcinoma cell line CaCo-2.

**Figure 3.**
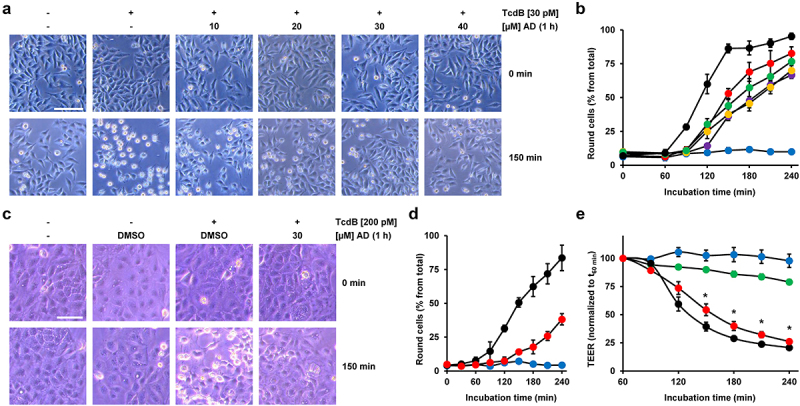
Effect of 1 h amiodarone preincubation on the intoxication of HeLa and CaCo-2 cells by TcdB. (a) HeLa cells were preincubated for 1 h with increasing concentrations of amiodarone (AD) as indicated or without amiodarone (− AD) and intoxicated with 30 pM TcdB (+ TcdB). Control cells were left without toxin treatment (− TcdB). Representative microscopic images are shown from time points 0 (upper row) and 150 min after TcdB intoxication (lower row). Scale bar represents 100 µm. (b) Graph shows the mean percentage values of round cells (in % from total cells) after quantification of TcdB (30 pM)-induced cell rounding over time in mock- (w/o amiodarone preincubation; black filled circles) and 1 h amiodarone-preincubated (10 µM, red filled circles; 20 µM purple filled circles; 30 µM, yellow filled circles; 40 µM green filled circles) HeLa cells. Blue filled circles represent the quantification of the percentage of cell rounding in HeLa cells without amiodarone preincubation and toxin addition. Mean percentage values of cell rounding were calculated from three parallel experiments in independent wells. Error bars represent ±SD. (c) CaCo-2 cells were preincubated for 1 h with 30 µM amiodarone (AD) or with solvent only (DMSO) prior to intoxication with 200 pM TcdB (+ TcdB). Control cells were left without toxin treatment (− TcdB). Representative microscopic images are shown from time points 0 (upper row) and 150 min after TcdB intoxication (lower row). Scale bar represents 100 µm. (d) Graph shows the mean percentage values of round cells (in % from total cells) after quantification of TcdB (30 pM)-induced cell rounding over time in mock- (w/o amiodarone preincubation; black filled circles) and 1 h amiodarone (30 µM)-preincubated (red filled circles) CaCo-2 cells. Blue filled circles represent the percentage of round CaCo-2 cells without amiodarone preincubation and toxin addition. Mean percentage values of cell rounding were calculated from three parallel experiments in independent wells. Error bars represent ±SD. (e) CaCo-2 cells grown in inserts to monolayers were preincubated for 1 h with 30 μM amiodarone (red circles) or with DMSO solvent (black circles), prior to apical addition of 200 pM TcdB and measurement of the transepithelial electrical resistance (TEER) every 30 min for up to 3 h. Parallel samples were not treated with TcdB (DMSO only, blue filled circles; amiodarone only, green filled circles). Diagram shows relative TEER values normalized to time point 60 min (t_60 min_; addition of the toxin). Error bars represent ±SEM (*n* = 8 in total from three independent experiments, black and red circles; *n* = 7 in total from three independent experiments, blue and green circles). Asterisks indicate statistical significance at each time point between red and black circles with *p < .05.

HeLa cells preincubated for 1 h either without or with increasing amiodarone concentrations were challenged with 30 pM TcdB for up to 4 h, prior to microscopic analysis and quantification of toxin-induced cell rounding over time. Importantly, also in HeLa cells, preincubation with 10 to 40 µM amiodarone for 1 h was sufficiently protective against TcdB-induced cell rounding. This is shown in representative microscopic images at time point 150 min after intoxication (Figure 3a), where TcdB-induced cell rounding was almost complete and the inhibitory effect of amiodarone most obvious. It is worth mentioning that HeLa cells exhibited a slightly faster response (~30 min earlier and with 30 pM instead of 60 pM TcdB) in terms of almost complete cell rounding upon intoxication with TcdB compared to Vero cells. The quantification of TcdB-induced cell rounding over time confirmed a delay of intoxication in amiodarone-preincubated cells (decreased cell rounding of about 50% at time point 150 min in cells preincubated for 1 h with 40 µM amiodarone) (Figure 3b).

Interestingly, robust inhibition of TcdB-induced cell rounding was also evident in CaCo2 cells preincubated for 1 h with 30 µM amiodarone, when compared to cells preincubated with solvent (DMSO) only Figure 3(c,d). To explore another approach for demonstrating that amiodarone can delay TcdB intoxication in CaCo-2 cells, we measured the transepithelial electrical resistance (TEER) across the cell monolayer. We exposed CaCo-2 cells growing in hanging cell culture inserts for 1 h to 30 μM amiodarone or only solvent (DMSO) before adding 200 pM TcdB at the apical side of the cells. Eventually, we recorded TEER every 30 min for up to 3 h after toxin addition. Amiodarone-preincubated cells showed a significantly delayed TEER drop than DMSO-preincubated cells after TcdB exposure (Figure 3e). This finding indicates that amiodarone helps in preserving the epithelial integrity of CaCo-2 cells against TcdB.

### Amiodarone preincubation protects Vero cells also from TcdA and the medically relevant combination of TcdA and TcdB

**Figure 4.**
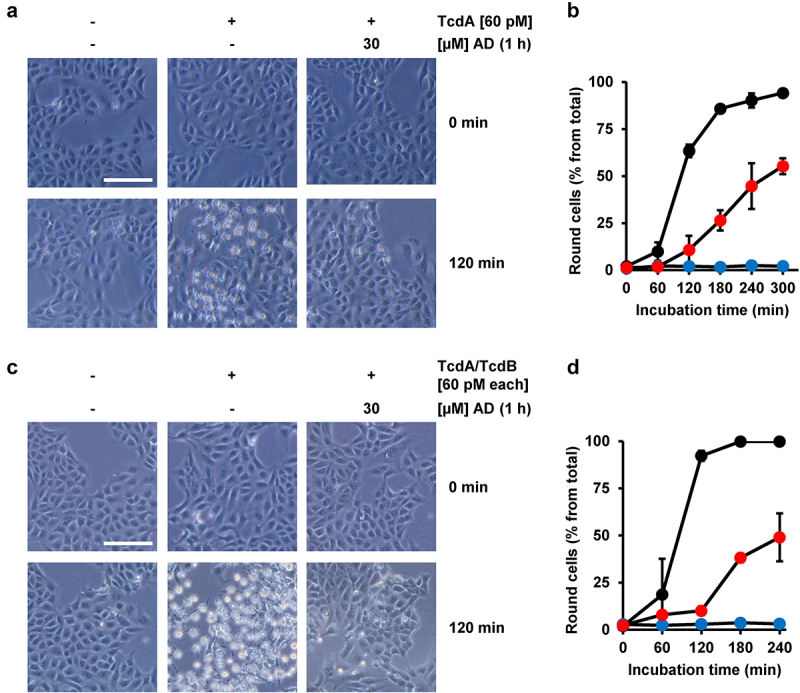
Effect of 1 h amiodarone preincubation on the intoxication of Vero cells by TcdA and the combination of TcdA and TcdB. (a,c) Vero cells were preincubated for 1 h with 30 µM amiodarone (AD), without amiodarone (− AD), prior to addition of (a) 60 pM TcdA (+ TcdA) or (c) the combination of 60 pM TcdA and 60 pM TcdB (TcdA/TcdB). Control cells in (a,c) were left without toxin treatment (- TcdA and – TcdA/TcdB, respectively) and without amiodarone treatment (− AD). Shown are representative microscopic images from time points 0 and 120 min after intoxication. Shown scale bar represents 100 µm. (b,d) quantification of cell rounding over time in mock- (black filled circles) and 1 h amiodarone-preincubated (30 µM, red filled circles) Vero cells intoxicated with (b) 60 pM TcdA or (d) the combination of TcdA and TcdB (60 pM each). Blue filled circles in (b,d) represent mean cell rounding percentage of Vero cells without amiodarone preincubation and toxin addition. Mean percentage values of round cells (in % from total cells) shown in (b,d) were calculated from three parallel experiments in independent wells. Error bars represent ±SD.

It has become clear in recent years that TcdA and TcdB do not share the same receptor specificity. Therefore, it was important to test whether amiodarone preincubation also delays the intoxication process of TcdA. To this end, Vero cells were preincubated for 1 h either with or without amiodarone, prior to intoxication with 60 pM TcdA. Importantly, microscopic analysis (Figure 4a) and quantification of TcdA-induced cell rounding over time (Figure 4b) revealed that TcdA intoxication was strongly delayed in the amiodarone-preincubated Vero cells. Moreover, we found that amiodarone preincubation of Vero cells for 1 h protected the cells against the combined toxic action of the medically relevant combination of TcdA and TcdB at 60 pM each Figure 4(c,d).

### Amiodarone-preincubated Vero cells are also less sensitive against TcdA and TcdB from the clinically relevant C. difficile strain NAP1/027

**Figure 5.**
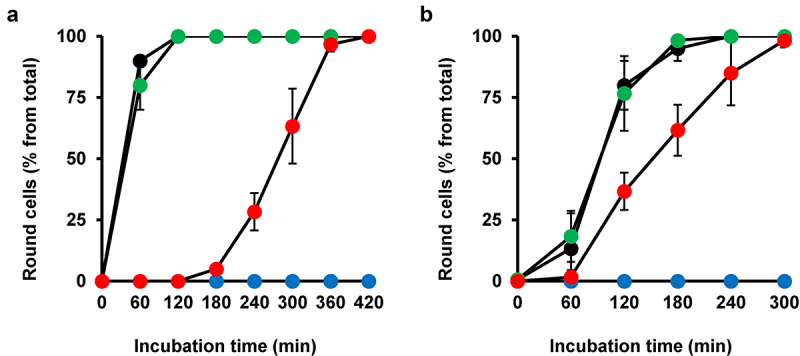
Effect of 1 h amiodarone preincubation on the intoxication of Vero cells by TcdA_NAP1_ and TcdB_NAP1_. (a,b) Vero cells were preincubated for 1 h with 10 µM amiodarone (red circles) or only with DMSO solvent (green circles) or were left without any pretreatment (black circles), prior to addition of (a) 600 pM TcdA_NAP1_ or (b) 60 pM TcdB_NAP1_ and incubation for up to (a) 420 min or (b) 300 min at 37°C. Mock-treated cells were left without toxin addition (blue circles). Quantification of cell rounding with mean percentage values of round cells (in % from total cells) is shown in (a,b), calculated from three parallel experiments in independent wells. Error bars represent ±SD.

Next, we wanted to clarify whether amiodarone can be used as a pan-variant inhibitor of TcdA and TcdB. Thus, we intoxicated Vero cells preincubated for 1 h with 10 µM amiodarone independently with both toxin variants isolated from the clinically relevant *C. difficile* strain NAP1/027. Intriguingly, quantification of toxin-induced cell rounding over time revealed for both toxin variants, TcdA_NAP1_ (Figure 5a) or TcdB_NAP1_ (Figure 5b), a robustly delayed intoxication of amiodarone-preincubated Vero cells, when compared to Vero cells without amiodarone preincubation. The inhibitory effect of amiodarone was even more pronounced against TcdA_NAP1_.

### Amiodarone does neither inhibit the intrinsic enzyme activities nor interfere with the receptor-binding domains of TcdB

**Figure 6.**
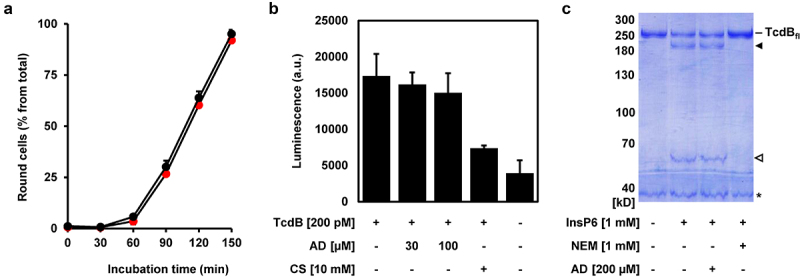
Amiodarone does neither inhibit intrinsic activities nor interfere with receptor-binding domains of TcdB. (a) TcdB (3 nM) was incubated without amiodarone (black circles) and with 30 µM amiodarone (red circles) in a total volume of 10 µl medium for 10 min at RT, followed by the addition to Vero cells growing in 500 µl medium in a 24-well (resulting in the dilution of TcdB to 60 pM and of amiodarone to sub-inhibitory 0.6 µM). Graph shows the mean percentage values of round cells (in % from total cells) after quantification of toxin-induced cell rounding over time. Mean percentage values of cell rounding were calculated from three parallel experiments in independent wells. Error bars represent ±SD. (b) UDP-Glo™ glycosyltransferase assay. 200 pM TcdB (+ TcdB) was either incubated with 30 or 100 µM amiodarone (AD) or with 10 mM castanospermine (CS) or without additional compounds (- AD; - CS). A sample without TcdB (− TcdB) and without any compounds (- AD; - CS) served as negative control for the assay. Bar diagram shows the mean luminescence in arbitrary units (a.u.), generated indirectly through the hydrolytic activity of the glucosyltransferase domain (GTD) of TcdB. Error bars represent ±SD of three reactions performed in parallel. (c) *in vitro* cleavage assay. TcdB (2 µg) was incubated for 1 h with 1 mM InsP6, either in the presence of 1 mM N-ethylmaleimide (NEM) or 200 µM amiodarone (AD) or without additional compounds (- NEM; - AD), followed by the visualization of the autoproteolytic fragments of TcdB by SDS-PAGE and Coomassie staining. A sample only with TcdB (- InsP6, - NEM, - AD) served as control for the non-processed, full-length TcdB (TcdB_fl_). TcdB fragment without GTD is indicated by a black filled arrow and the GTD by a black open arrow. Asterisk indicates a protein impurity in the TcdB preparation.

TcdA and TcdB, and their variants use different host receptors for cell entry. It is thus unlikely that the above mentioned findings are explained by an influence of amiodarone on all the individual interactions between the various toxins and their specific receptors. This is confirmed by our finding that preincubation of TcdB (3 nM) with amiodarone (30 µM) in 10 µl medium for 10 min at room temperature, prior to the addition of Vero cells growing in 24-wells (500 µl medium; hereby TcdB is diluted to 60 pM and amiodarone to sub-inhibitory 0.6 µM), did not inhibit TcdB-induced cell rounding (Figure 6a). Conclusively, amiodarone does not interfere with the receptor-binding domains of TcdB prior to binding and entry into host cells via receptor-mediated endocytosis.

Instead, it seems that a more central process required for the uptake and/or mode-of-action of both toxins, TcdA and TcdB, including their variants, is inhibited by amiodarone. We therefore aimed to get further insights into the underlying inhibitory mode-of-action of amiodarone and analyzed *in* *vitro* the enzymatic activities of the glucosyltransferase and/or cysteine protease domain of TcdB.

The glucosyltransferase activity of TcdB was analyzed by performing a UDP-Glo™ Glycosyltransferase Assay. Castanospermine, a compound described as a potent inhibitor of the GTD of TcdB, was used as positive control in this assay.^14,24^ Whereas 10 mM castanospermine showed, as expected, robust inhibition of the glucosyltransferase activity of TcdB (~50% reduction), the same approach with 30 and 100 µM amiodarone yielded no inhibition (Figure 6b).

The autoproteolytic activity of the CPD of TcdB was analyzed by incubating native, full-length TcdB protein with 1 mM InsP6, either in the presence or absence of 200 µM amiodarone or 1 mM of the well-accepted cysteine protease inhibitor NEM (N-ethylmaleimide),^25^ followed by SDS-PAGE and Coomassie staining for visualizing the autoproteolytic cleavage of the GTD from the holotoxin. Whereas NEM was able to fully block InsP6-activated cleavage of the GTD from the TcdB holotoxin, amiodarone had absolutely no effect on this process (Figure 6c).

In summary, inhibition of the intrinsic enzyme activities of TcdB seems not to be the inhibitory mode-of-action of amiodarone that leads to the protection of cells from TcdB intoxication.

### Intracellular accumulation of amiodarone is a prerequisite for inhibition of TcdB upon cell entry

By performing a series of experiments, we sought out to unravel the mechanism behind amiodarone’s inhibition of TcdB. At first, we asked whether amiodarone is capable of inhibiting TcdB in Vero cells even without a preincubation step with the compound. We therefore preincubated Vero cells for increasing time periods with 30 µM amiodarone and removed amiodarone by medium exchange prior to intoxication with 60 pM TcdB. Amiodarone removal by medium exchange was required, in order to avoid the accumulation of amiodarone from the medium into the cells during the incubation period with TcdB. Toxin-induced cell rounding was then analyzed microscopically over time.

**Figure 7.**
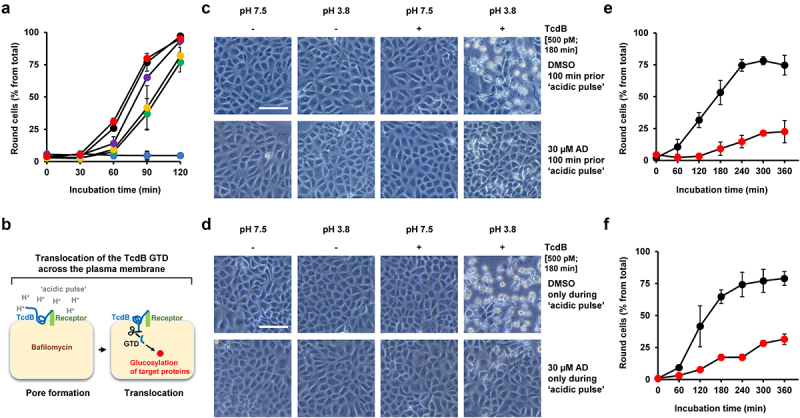
Impact of the preincubation period of amiodarone on TcdB intoxication of Vero cells and effect of amiodarone on TcdB-intoxicated cells directly at the plasma membrane. (a) Vero cells were preincubated for increasing time periods with 30 µM amiodarone, followed by removal of amiodarone (AD) by medium exchange prior to intoxication with 60 pM TcdB. Graph shows the mean percentage values of round cells (in % from total cells) after quantification of toxin-induced cell rounding over time. Amiodarone preincubation was 0 min for red filled circles, 20 min for purple filled circles, 40 min for yellow filled circles, and 60 min for green filled circles. Black filled circles indicate cells incubated with TcdB but without amiodarone and blue filled circles represent cells incubated without amiodarone and TcdB. Mean percentage values of cell rounding were calculated from three parallel experiments in independent wells. Error bars represent ±SD. (b) illustration of the TcdB intoxication of cells at the plasma membrane by an ‘acidic pulse’. The cells are preincubated with bafilomycin A1, an inhibitor of endosomal acidification, and kept at 4°C during incubation with the toxin to inhibit endocytosis. Next, the growth medium of the cells is exchanged with ‘low pH-medium’ (pH 3.8). During this step (‘acidic pulse’), the cell surface-bound TcdB molecules insert into the plasma membrane and translocate their GTD directly from here into the cytosol, thus bypassing the endosomes. Eventually, the cells are kept again at neutral pH and the intoxication (cytopathic cell rounding), starting from here (time point 0 min), is monitored over time microscopically. As a negative control, cells are kept in parallel in medium without an ‘acidic pulse’ (pH 7.5), where translocation of the GTD across the plasma membrane, and consequently toxin-induced cell rounding, is prevented. The detailed procedure is described in the materials and methods section. (c,d) experiment was performed as described in (b) with 500 pM TcdB (+ TcdB), but in (c) with 100 min incubation in total with 30 µM amiodarone (lower row) or DMSO solvent (upper row) prior the ‘acidic pulse’ and in (d) with incubation of the cells with 30 µM amiodarone (lower row) or with DMSO (upper row) only during the ‘acidic pulse’. Controls without TcdB addition were performed in parallel (− TcdB). Shown exemplary images were obtained microscopically at time point 180 min after intoxication. Scale bar represents 100 µm. (e,f) quantification of TcdB-induced cell rounding at indicated timepoints after intoxication by an ‘acidic pulse’ (pH 3.8) in cells shown in (b) after DMSO (black filled circles) or AD-incubation (red filled circles) and in (c) with DMSO (black filled circles) or AD (red filled circles) in the ‘acidic pulse’ medium, respectively. Mean percentage values of cell rounding were calculated from three parallel experiments in independent wells. Error bars represent ±SD.

Interestingly, as shown in Figure 7a, inhibition of TcdB intoxication after preincubation of cells for 1 h with amiodarone was also observed after removal of amiodarone by medium exchange prior to addition of TcdB. However, the preincubation period with amiodarone had an impact on the extent of cell rounding after intoxication with TcdB. The shorter the preincubation period with amiodarone, the smaller was the inhibitory effect of amiodarone on TcdB intoxication (Figure 7a). These observations led us assume that an intracellular step during TcdB intoxication, which requires preceding cytosolic accumulation of amiodarone, is influenced by amiodarone.

### Amiodarone interferes with the translocation pore of TcdB

Amiodarone is a multichannel blocker and thus might inhibit vesicular H^+^-ATPases (V-ATPases) that function as ATP-driven proton pumps in endosomes. Endosomal acidification by V-ATPases is crucial for the translocation of TcdB, including low pH-mediated membrane insertion, pore formation and transport of the GTD into the cytosol. To test whether amiodarone preincubation of Vero cells inhibits endosomal acidification, LysoTracker Green DND-26, a cell-permeable, green fluorescent dye that stains acidic compartments within a cell, was employed. LysoTracker-stained intracellular vesicles were readily visible in DMSO (solvent) but also in amiodarone (30 µM, 1 h)-preincubated Vero cells. As expected, an almost complete loss of LysoTracker-stained intracellular vesicles was observed in Vero cells preincubated for 1 h with 200 nM bafilomycin A1, an established inhibitor of V-ATPases and thus of endosomal acidification (Supplementary Fig. S1). Based on these results, it is unlikely that amiodarone inhibits endosomal acidification within 1 h to such an extent that would explain its inhibitory potential against TcdA and TdcB intoxication.

We then asked whether amiodarone inhibits TcdB intoxication at conditions where the toxin bypasses the endocytic route into cells. To this end, we intoxicated cells with TcdB either in the presence or absence of amiodarone directly at the plasma membrane. This well-established intoxication procedure is described and illustrated in Figure 7b. Shortly, an ‘acidic pulse’ forces the cell surface-bound TcdB to insert into the plasma membrane and to translocate its GTD into the cytosol directly from here, thus bypassing the endosomal route for target substrate modification.

To our surprise, TcdB intoxication of Vero cells by this approach was readily inhibited either when cells were incubated prior to the ‘acidic pulse’ with 30 µM amiodarone for 100 min in total (Figure 7c,e) or at conditions where the same concentration of amiodarone was solely present in the low pH-medium only during the ‘acidic pulse’ (Figure 7d,f). Importantly, quinacrine, a lysosomotropic agent capable of inhibiting endosomal acidification, did not decrease TcdB-induced cell rounding when incubated at a concentration of 5 µM prior to the ‘acidic pulse’ for 100 min in total (Supplementary Fig. S2). We have chosen 5 µM quinacrine as optimal concentration for this assay, since it has been shown previously that preincubation of Vero cells for 1 h already with 3.3 µM quinacrine was sufficient to prevent SARS-CoV-2 infection (99% inhibition; IC_50_: 1.373 µM) by its ability to raise pH in endocytic vesicles.^26^ This control experiment was important, since it might be feasible that although cells were preincubated with bafilomycin A1, the endocytic route was not entirely blocked for toxin entry. Under such a scenario, it is conceivable that amiodarone, if still considered to inhibit V-ATPases under these experimental conditions, merely inhibited endosomal acidification and not the translocation of TcdB across the plasma membrane. However, no inhibitory effect of quinacrine in this context indicates that the endocytic route was efficiently sealed with bafilomycin A1. Thus, the inhibitory effect of amiodarone when added prior to the ‘acidic pulse’ points toward a direct interference of the inhibitor with the translocation pore of TcdB.

From these findings, we conclude that amiodarone’s mode of TcdB inhibition is not solely explained at the level of endocytic uptake and/or by direct inhibition of endosomal V-ATPases. The immediate inhibitory effect of amiodarone on plasma-membrane-associated TcdB hints toward an additional role of amiodarone as an inhibitor of the translocation pore during TcdB’s intoxication process.

### Amiodarone inhibits Rac1 modification by TcdB in a human ‘minigut’ model

**Figure 8.**
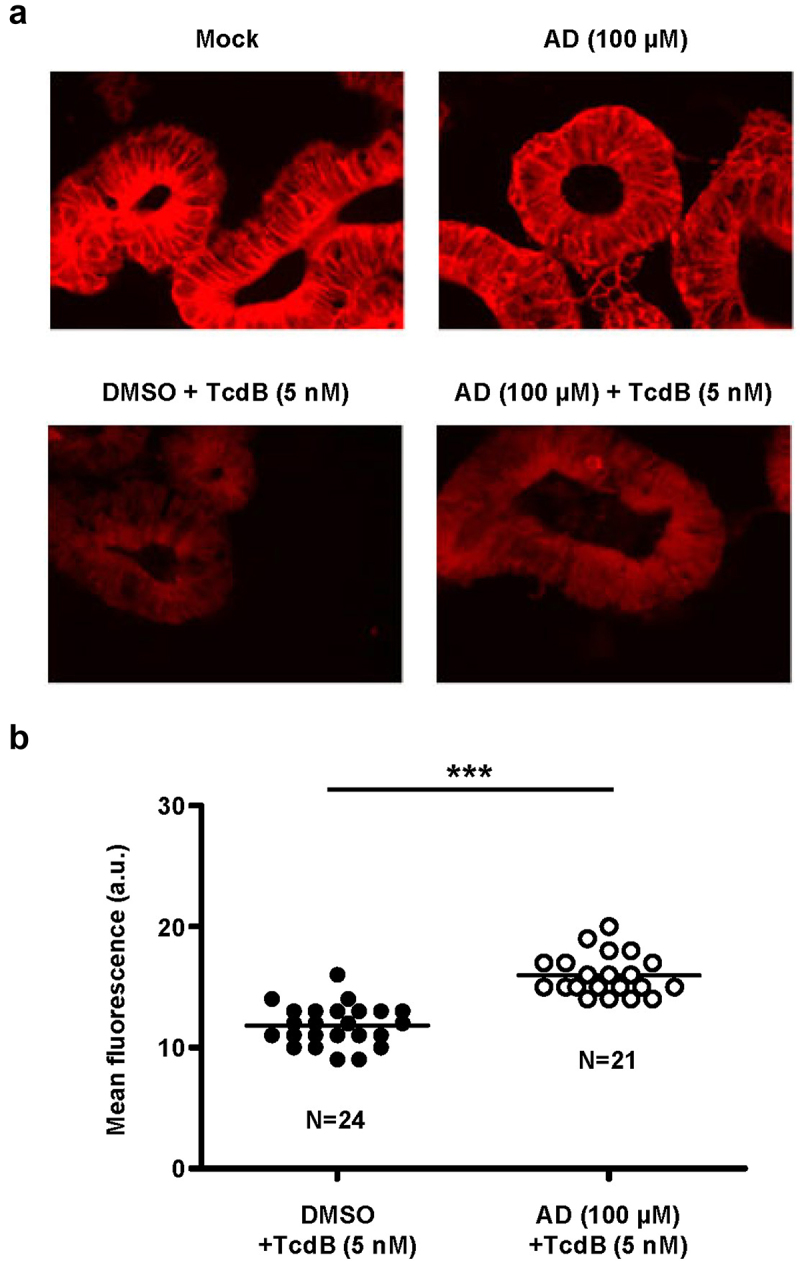
Effect of 24 h amiodarone preincubation of human ‘miniguts’ on Rac1 modification by TcdB. (a) human ‘miniguts’ were preincubated for 24 h with 100 µM amiodarone (AD) or with DMSO solvent, prior to the incubation with 5 nM TcdB for 3 h and followed by Rac1 immunostaining (Alexa Fluor® 633) and immunofluorescence microscopy with a 40× oil objective. In parallel samples, control cells, either untreated (mock) or pretreated for 24 h with AD (AD (100 µM)), were left without TcdB intoxication. Exemplary fluorescence microscopy images are shown that were obtained 3 h after TcdB intoxication. Scale bar represents 50 µm. (b) scatter dot plot shows the quantification of the mean fluorescence intensity (in arbitrary units, a.u.) of Rac1 signals from TcdB-intoxicated ‘miniguts’ that were either preincubated with DMSO (blacked filled dots) or with amiodarone (white filled dots) as described in (a). Each dot represents one image that was used for the quantification of the mean Rac1 signal intensity in the ‘miniguts’, with N representing the total amount of analyzed images per condition and the horizontal line representing the mean of all individual mean fluorescence intensities per image and condition. Asterisk indicates statistical significance between two groups with ***p < .001.

Finally, we were keen to show that amiodarone is capable of inhibiting TcdB in the clinically relevant human ‘minigut’ model, represented by stem cell-derived 3D human intestinal organoids that reproduce numerous crucial characteristics of typical colonic epithelium. For that purpose, ‘miniguts’ were preincubated for 24 h with 100 µM amiodarone or with DMSO solvent only, prior to intoxication with 5 nM TcdB for 3 h and followed by Rac1 immunostaining and fluorescence microscopy. As shown above in Figure 2, an anti-Rac1 antibody was used for immunostaining that recognizes only non-glucosylated Rac1. Consequently, in TcdB-treated organoids, Rac1 fluorescence signals were markedly decreased due to cell entry and action of the toxin, when compared to mock-treated cells (Figure 8a). Representative fluorescence microscopy images of the ‘miniguts’ after TcdB intoxication and Rac1 immunostaining are shown in Figure 8a. Importantly, Rac1 fluorescence signals after TcdB intoxication were significantly increased in amiodarone-preincubated ‘miniguts’, in direct comparison to ‘miniguts’ preincubated with DMSO only (Figure 8a,b). The inhibitory potential of amiodarone against TcdB intoxication of ‘miniguts’ was confirmed by repeating the experiment with ‘miniguts’ preincubated for 24 h with 250 µM amiodarone followed by intoxication with 3 nM TcdB for 3 h (Supplementary Fig. S3).

Overall, our study provides convincing evidence that the already licensed antiarrhythmic drug amiodarone represents a potent pan-variant inhibitor of the *C. difficile* toxins TcdA and TcdB and might be thus repurposed in the future as an antitoxin therapeutic in the context of *C. difficile*-associated diseases (CDADs).

## Discussion

Although the exact role of membrane cholesterol during the cellular uptake of TcdA and TcdB has not been fully understood, it seems that efficient membrane insertion and/or pore formation of the toxins in endosomal membranes requires the presence of cholesterol.^17^ It is feasible that membrane cholesterol assists in the correct positioning of transmembrane segments of these toxins within endosomal membranes for generating a functional translocation pore. Consequently, membrane cholesterol-dependence of TcdA and TcdB represents a promising Achilles’ heel for therapeutic targeting of both toxins.

Several membrane cholesterol-lowering strategies have been applied so far in order to inhibit the intoxication of target cells by TcdA and TcdB: i) inhibition of cholesterol uptake and/or biosynthesis with the hypocholesterolemic drug simvastatin or compounds such as PF-429242 and 25-hydroxycholesterol acting on the SREBP-2 pathway,^18^ ii) inhibition of intracellular distribution and transport of cholesterol with the compound U18666A,^19^ and iii) depletion of cholesterol from membranes with methyl-beta-cyclodextrin.^17^ For the antiarrhythmic drug amiodarone, it has been shown recently that it is able to decrease cholesterol content in cellular membranes.^21–23^ This previously rather unknown effect of amiodarone on cells seems to be related to the inhibition of a specific enzyme involved in cholesterol biosynthesis, namely the 24-dehydrocholesterol reductase.^21,22^ Thus, we were keen to test in this study whether preincubation of cultured cells with amiodarone protects from TcdA and/or TcdB intoxication.

Starting with prototypical TcdB from the historical *C. difficile* strain VPI 10463 as representative for both glucosylating toxins, we could undoubtedly show that amiodarone renders African green monkey-derived Vero cells, human HeLa and CaCo-2 cells as well as human intestinal organoids less sensitive toward the toxin. In addition, we could confirm in Vero cells the protective effect of amiodarone against TcdA, the medically relevant combination of TcdA and TcdB as well as against TcdA and TcdB variants isolated from the epidemic *C. difficile* strain NAP1/027. Interestingly, TcdA from both strains was more efficiently inhibited by amiodarone when compared to its TcdB counterparts.

Just recently, the membrane cholesterol-decreasing feature of amiodarone was found to exert an antiviral effect, also against SARS-CoV-2.^23^ Sanchez and colleagues have shown that in addition to viruses, certain bacterial toxins, such as the anthrax and the diphtheria toxin, are also inhibited by amiodarone.^27^ It was hypothesized by the authors that amiodarone’s inhibitory mode-of-action relies on neutralization of endosomal pH, thereby blocking cell entry of the toxins. This is in line with the observation of Piccoli et al., who found no inhibitory effect of amiodarone on Shiga toxin, which belongs to the so-called ‘long-trip toxins’.^28^ This sub-group of AB-type bacterial toxins enters cells via retrograde trafficking through the secretory pathway and independent of endosomal acidification.^29^

TcdA and TcdB are ‘short-trip toxins’, such as the anthrax and the diphtheria toxin, which translocate their enzyme portions into the host cell cytosol directly from early and/or late endosomes. As a prerequisite, acidification of the endosomes via V-ATPases is needed, for proper insertion and pore formation of the toxins into the endosomal membrane.^11,12^ Our data argue against but do not exclude the possibility that amiodarone inhibits TcdA/TcdB intoxication via inhibition of endosomal acidification as it has been suggested for the anthrax and the diphtheria toxin.^27^ However, additional effects of amiodarone, such as the recently described decrease of the cholesterol content in membranes, might contribute to the inhibitory potential against TcdA and TcdB. Supportively, Vero cells preincubated for 24 h with amiodarone exhibited longer protection times against TcdB intoxication when compared to 1 h preincubation.

Strikingly, our experimental data point toward an additional inhibitory mechanism of amiodarone against TcdA and TcdB, namely, interference with the translocation pore of the toxins. Our hypothesis is supported by the fact that TcdB, which we forced via an ‘acidic pulse’ to translocate its enzyme portion into the cytosol at the plasma membrane and thus by evading the endosomal route, was also readily inhibited by amiodarone, which was either preincubated with the cells or directly added from outside to cells with the ‘acidic pulse’ medium. In this context, immediate inhibition of TcdB by amiodarone can only be explained by insertion of the compound into and blockage of the translocation pore, either from inside or outside the plasma membrane, and interference with the translocation of the glucosyltransferase domain of the toxin into the cytosol. Our data exclude the possibility that amiodarone delays TcdB intoxication of cells by inhibiting the enzyme activities of its glucosyltransferase and cysteine protease domain, which are relevant after entry of the toxin into host cells, or by interfering with its receptor-binding domains.

Amiodarone (chemical structure is provided in Supplementary Fig. S4) is a multichannel inhibitor, which belongs primarily to the class III antiarrhythmic drugs characterized as potassium channel blockers. In addition, amiodarone is also inhibiting cardiac sodium and calcium channels.^20,30–33^ Remarkably, the translocation pores of TcdA and TcdB have been shown to be permeable for monovalent cations, such as potassium and rubidium ions.^34^ Thus, it is quite feasible that amiodarone is capable of entering into and clogging of the translocation pore of TcdA and TcdB, which might share structural similarities with the inner pore of potassium channels. Further studies are needed to elucidate the molecular details of the interference of amiodarone with the translocation pore of TcdA and TcdB. Our attempts to address this question by performing ‘black’ lipid membrane measurements failed due to the inherent effects of amiodarone on the ‘painted’ artificial lipid bilayer (data not shown).

Due to interstrain heterogeneity in the genes encoding TcdA and TcdB, resulting in several TcdA and TcdB variants, strategies for the pan-variant inhibition of TcdA and TcdB are likely to gain increased medical interest in the near future. For instance, bezlotoxumab, the only FDA-approved therapeutic antibody targeting VPI 10463-derived TcdB, is much less potent on neutralizing NAP1-derived TcdB.^35,36^

Therefore, drug repurposing of amiodarone, an already licensed antiarrhythmic agent, might represent a safe antitoxin strategy for the (supportive) therapy of severe *C. difficile*-associated diseases (CDADs) caused by epidemic and/or hypervirulent strains. Indeed, the steady-state concentrations of amiodarone in the plasma were found to lie between 0.4 and 11.99 μg/ml (~0.6 to ~19 µM) and are thus in a similar range of the concentrations of amiodarone used in our study.^37^ Notably, amiodarone, owing to its extracardiac toxicity, can have serious side effects (15% prevalence in the first year and up to 50% with long-term use).^38–40^ However, in the treatment of severe CDADs, a rather short-term use and topical rectal low-dosage administration of amiodarone is conceivable, which might limit the risk of serious side effects during therapy. Fortunately, dronedarone, an already FDA-approved amiodarone analogue with a significantly improved side effect profile, is nowadays available.^41,42^ In future studies, we want to unravel whether dronedarone is also capable of inhibiting TcdA/TcdB intoxication and thus might serve as safer-to-use alternative to amiodarone.

## Materials and methods

### Cell culture

HeLa and Vero cells were grown in Minimum Essential Medium (MEM; Fisher Scientific GmbH, Schwerte, Germany; #11524426) supplemented with 10% fetal calf serum (FCS), 1% sodium pyruvate, 1% non-essential amino acids, 1% penicillin/streptomycin, and 2 mM L-glutamine.

CaCo-2 cells were cultivated in Dulbecco’s Modified Eagle’s Medium (DMEM; Fisher Scientific GmbH, Schwerte, Germany; #11594486) supplemented with 10% FCS, 1% sodium pyruvate, 1% non-essential amino acids, and 1% penicillin/streptomycin.

All cell lines were maintained in the incubator under humidified conditions at 37°C and 5% CO_2_.

### Cytopathic cell rounding assay

For performing cytopathic cell rounding assays with TcdA and/or TcdB, HeLa and/or Vero cells were typically seeded into wells of 24-well plates and grown until they reached subconfluency. Toxins were added, as indicated, either directly into the medium or the medium of each well was exchanged with toxin-containing medium, followed by further incubation of the well plates at 37°C in the incubator. Optionally, cells were preincubated with growth medium supplemented with amiodarone or solvent control (DMSO), as indicated. Toxin-induced cell rounding was quantified manually in microscopic images by using the Neuralab online tool (https://neuralab.de).

### Intoxication of cultured cells by TcdB at the plasma membrane

The intoxication of Vero cells with TcdB at conditions directly at the plasma membrane, through which the toxin bypasses the endocytic route, was performed as follows: i) inhibition of endosomal acidification: exchange of the growth medium of nearly confluent Vero cells in wells of a 24-well plate with pre-warmed (37°C) growth medium including 200 nM bafilomycin A1 (‘baf-medium’) and incubation for 30 min at 37°C; ii) toxin binding to the cell surface: incubation of the cells for 10 min at 4°C followed by the exchange of the ‘baf-medium’ with ice-cold ‘baf-medium’ including 500 pM TcdB (‘tox medium’) and incubation of the cells for 30 min at 4°C; iii) ‘acidic pulse’ for triggering plasma membrane-insertion of the toxin and translocation of the GTD into the cytosol: removal of the ‘tox medium’ and drop-wise addition of pre-warmed (37°C), serum-free growth medium adjusted to pH 3.8 (‘low pH-medium’) to the cells followed by incubation for 10 min at 37°C; iv) starting time point of the intoxication: exchange of the ‘low pH-medium’ with pre-warmed (37°C) ‘baf-medium’ (pH 7.5) and microscopical monitoring of cytopathic cell rounding over time. As negative control, regular serum-free growth medium with neutral pH (pH 7.5) was used instead of ‘low pH-medium’ for preventing the translocation of the GTD across the plasma membrane and toxin-induced cell rounding.

Two options were used for testing the inhibitory potential of amiodarone on TcdB intoxication of cells at the plasma membrane. The first option was to preincubate Vero cells for 30 min with 30 µM amiodarone (or DMSO solvent) followed by further incubation of the cells with amiodarone by including the compound in the ‘baf-medium’ (step i) and ‘tox medium’ (step ii). This resulted in a total incubation period of the cells with the compound of 100 min, prior to the ‘acidic pulse’ (step iii) and the starting time point of the intoxication (step iv). For the second option, amiodarone was added only to the ‘low pH-medium’ (step iii).

### Human intestinal organoids

Human intestinal organoids (HIOs, ‘miniguts’) were received from the Core Facility Organoids from the Ulm University, headed by Alexander Kleger, and incubated in Matrigel and medium as described elsewhere.^43,44^ The use of the human material in this study is in compliance with the guidelines of the Federal Government of Germany and the Declaration of Helsinki concerning Ethical Principles for Medical Research Involving Human Subjects and has been approved by the ethical committee of the Ulm University (No. 0148/2009) and Tübingen University (638/2013BO1). Intoxication experiments with ‘miniguts’ and subsequent analysis of Rac1 modification via Rac1 immunostaining and fluorescence microscopy were performed in 48-well plates essentially as described previously.^45,46^

### Toxins, inhibitors and other reagents

TcdA and TcdB from *C. difficile* NAP1/027 strain were purified as described previously from 72 h culture supernatants using dialysis culture in Brain Heart Infusion broth^47,48^. TcdB from *C. difficile* VPI 10463 was a generous gift from Klaus Aktories (University of Freiburg, Germany) and purified as described before.^49^

Amiodarone hydrochloride was ordered from Thermo Fisher Scientific (Waltham, USA; J60456) or from Supelco (Bellefonte, USA; PHR1164), dissolved in dimethyl sulfoxide (DMSO) to obtain a 10 mM stock solution, which was stored at -20°C. Bafilomycin A1 (sc-201550; 100 µM in DMSO), inositol hexakisphosphate (InsP6; sc-250718; stock: 100 mM in H_2_O) and castanospermine (sc-201358; stock: 100 mM in H_2_O) were from SantaCruz Biotechnology (Dallas, USA). N-ethylmaleimide (NEM) and quinacrine were ordered from SigmaAldrich. LysoTracker™ Green DND-26 and Hoechst 33342 were obtained from Thermo Fisher Scientific.

### Microscopy

The following microscopes were used for analyzing the morphology of cell monolayers: Leica DMi1 equipped with a Leica MC170 HD or Leica Flexacam C1 camera (Leica, Wetzlar, Germany); Eclipse 80i (Nikon, Tokyo, Japan).

Fluorescence microscopic images from immunostained cultured cells and human intestinal organoids were obtained with an iMIC digital microscope (Till Photonics, Gräfelfing, Germany; provided by the group of Manfred Frick from the Institute of General Physiology at the University of Ulm) equipped with an oligochrome light source (390/40 nm excitation filter for Hoechst 33342, 482/20 nm excitation filter for phalloidin-FITC, 640/20 nm excitation filter for Alexa Fluor® 633, 563/20 nm excitation filter for Alexa Fluor® 568), a quadband filter for standard ICC/IHC applications and TIRF, and a CCD Clara camera (Andor). Image processing (cropping; enhancement of brightness and/or contrast) was performed with ImageJ, GIMP or with Microsoft PowerPoint. ImageJ was used for the quantification of the mean fluorescence signal intensity of each organoid.

Fluorescence microscopic images from Vero cells stained with LysoTracker Green DND-26 for visualization of acidic compartments were obtained with the BZ-X810 All-in-One fluorescence microscope (Keyence, Neu-Isenburg, Germany), equipped with a Plan Apochromat 40X objective and BZ-X filters for DAPI (OP-87762) and GFP (OP-87763).

### Lysotracker staining of acidic compartments in Vero cells

For staining intracellular acidic compartments in Vero cells, LysoTracker Green DND-26 was added from a 10 mM stock solution in DMSO at a final concentration of 50 nM directly to the medium, and cells were further incubated for 10 min at 37°C, followed by fluorescence microscopical analysis. Cell nuclei were stained with Hoechst 33342.

### Preparation of whole-cell lysates and Rac1 immunoblotting

For the generation of whole-cell lysates from cultured cells growing in wells of 24-well plates, at first the growth medium was removed and then the cell monolayers were frozen and thawed again by resuspension in 2.5-fold pre-heated (95°C) Laemmli buffer. Cell lysates were then heated for 10 min at 95°C, followed by SDS-PAGE and transfer of the lysate proteins by Western blotting onto a nitrocellulose membrane. For the immunodetection of non-glucosylated Rac1 and GAPDH, primary mouse anti-Rac1 (#610651; clone 102; BD Biosciences, Heidelberg, Germany) and mouse anti-GAPDH (sc-365062; G-9; Santa Cruz Biotechnology, Dallas, USA) antibodies were used, respectively. Horseradish peroxidase (HRP)-coupled mouse IgG kappa-binding protein (m-IgGκ BP-HRP; Santa Cruz Biotechnology, Dallas, USA; sc -516,102) and HRP-coupled goat anti-mouse IgG (H+L) secondary antibody (#31430; Thermo Fisher Scientific, Waltham, USA) were used for the generation of antibody signals that were detected by the enhanced chemiluminescence (ECL) reaction.

### UDP-Glo™ glycosyltransferase assay

To monitor the activity of the glucosyltransferase activity of TcdB, the UDP-Glo™ Glycosyltransferase Assay (V6991; Promega, USA) was used, following the manufacturer’s recommendations. The reaction was performed with UDP-glucose as co-substrate and with the GTPase Rac1 as substrate, which was recombinantly purified from *Escherichia coli* as described previously.^24^ In brief, all reactions were performed for 1 h at 37°C in a total volume of 15 µl of glucosylation buffer (50 mM HEPES, 100 mM KCl, 2 mM MgCl_2_, 1 mM MnCl_2_, 100 mg/L BSA, pH 7.5), including 200 pM TcdB, 100 µM UDP-glucose, 5 µM Rac1, and, optionally, amiodarone (30 or 300 µM) or castanospermine (10 mM). Next, 10 µl of each reaction was transferred to a 96-well half-area microplate (Greiner, #675075, Austria). Reactions were then stopped by addition of 10 µl UDP Detection Reagent, followed by shaking at 1,000 rpm for 30 s. Luminescence signals were recorded using a Tecan infinite M1000Pro plate reader (Tecan Trading AG, Switzerland) with an adjusted integration time of 750 ms.

### InsP6-induced autoproteolytic cleavage of TcdB

TcdB (2 µg) was incubated with 1 mM InsP6 for 1 h at 37°C in a total volume of 20 µl, buffered with 20 mM Tris and 150 mM NaCl at pH 7.4. Reaction was stopped by the addition of 5 µl 5× Laemmli buffer followed by heating for 10 min at 95°C. Autoproteolytic cleavage products of TcdB were analyzed by SDS-PAGE and Coomassie staining of the gel.

### Measurement of transepithelial electrical resistance

We used the EVOMX apparatus equipped with the STX2 electrode (World Precision Instruments, Sarasota, United States) for measuring the transepithelial electrical resistance (TEER) of CaCo-2 cells. At day 0, 1.5 × 10^5^ cells were seeded into 24-well hanging cell culture inserts (Brand GmbH, Wertheim, Germany; polyester membrane, cellGrade plus, pore size 0.4 μm) and incubated at 37°C until day 3, where the cells showed initial TEER values typically between ~1300 and ~1800 Ω. Then, amiodarone (30 µM) or solvent (DMSO) was added apically and basolaterally to the cells. Following 1 h of incubation at 37°C, TcdB (200 pm) was added apically to initiate the intoxication of the cells. TEER was measured every 30 min for up to 3 h after toxin addition. TEER values were normalized to time point 60 min (t_60 min_; addition of the toxin) and set to 100%. Parallel samples without TcdB addition were included as controls.

### Statistics

All experiments were performed at least twice, either with identical or slightly modified conditions (‘miniguts’ experiments) or with at least three independent replicates (e.g. three independent wells) per experiment for generating diagrams for the cytopathic cell rounding assays. Unpaired t-test was used for calculating the significance of differences between mean values of two or more groups. Resulting p-values were indicated directly in diagrams by asterisks as follows: *p < .05, **p < .01, ***p < .001.

## Supplementary Material

Supplemental Material

## Data Availability

The data that support the findings of this study are openly available in RADAR4Chem at https://dx.doi.org/10.22000/942.
