# Supplementary material for: Exploring the inhibitory potential of the antiarrhythmic drug amiodarone against Clostridioides difficile toxins TcdA and TcdB
Source: Gut Microbes. 2023 Sep 25;15(2):2256695. doi: 10.1080/19490976.2023.2256695 (PMC10524773; doi:10.1080/19490976.2023.2256695)
Supplement: Supplemental Material [file KGMI_A_2256695_SM0173.docx]

**Supplementary Figures**

For manuscript entitled “**Exploring the Inhibitory Potential of the Antiarrhythmic Drug Amiodarone Against *Clostridioides difficile* Toxins TcdA and TcdB**” by Schumacher et al.


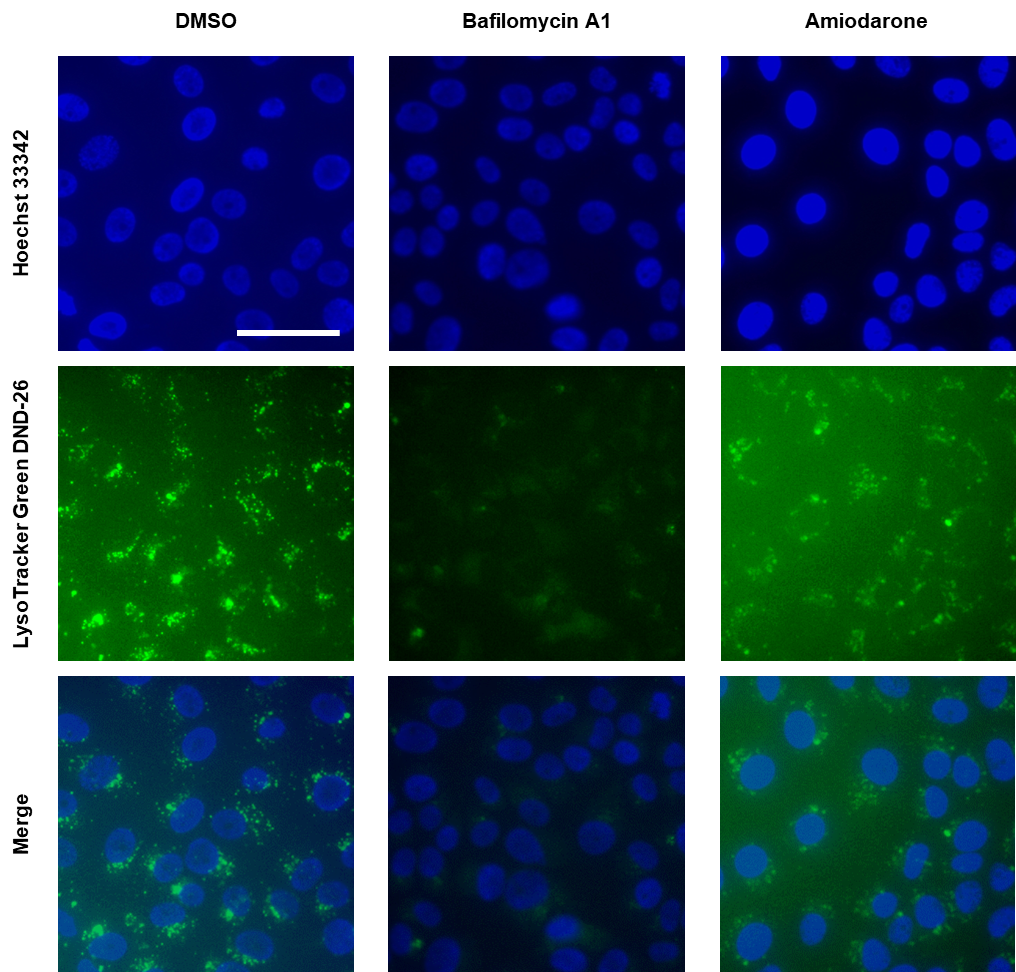


**Supplementary Figure 1.** Influence of amiodarone-preincubation on acidification of endocytic vesicles in Vero cells. Vero cells were preincubated for 1 h at 37°C either with 200 nM bafilomycin A1 (positive control; established inhibitor of endosomal acidification), 30 µM amiodarone or only solvent (DMSO), followed by incubation of the cells for 10 min at 37°C with 50 nM LysoTracker Green DND-26 and visualization of acidic compartments (shown in green; middle row) and Hoechst 33342-stained cell nuclei (shown in blue; upper row) by fluorescence microscopy. Merge (lower row) represents the overlay of both images. Scale bar indicates 50 µm.


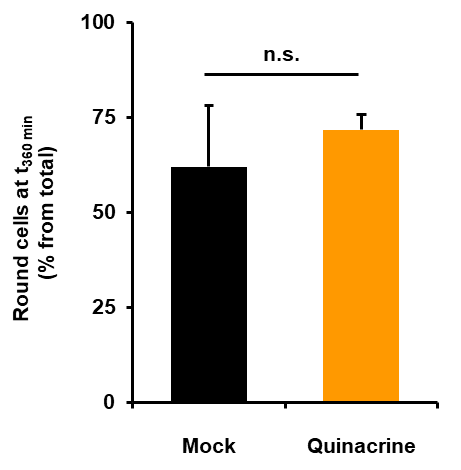


**Supplementary Figure 2.** Influence of the preincubation of Vero cells with the lysosomotropic compound quinacrine on TcdB-intoxication directly at the plasma membrane. Experiment was performed as described in Figure 7B with 500 pM TcdB and with 100 min incubation in total with 5 µM quinacrine or only medium (mock) prior the ‘acidic pulse’. Bar diagram shows the quantification of TcdB-induced cell rounding at timepoint 360 min (t_360 min_) after intoxication by an ‘acidic pulse’ (pH 3.8), with black bar indicating mock- and orange bar indicating quinacrine-preincubated Vero cells. Mean percentage values of cell rounding were calculated from three parallel experiments in independent wells. Error bars represent ±SD. n.s., not significant.


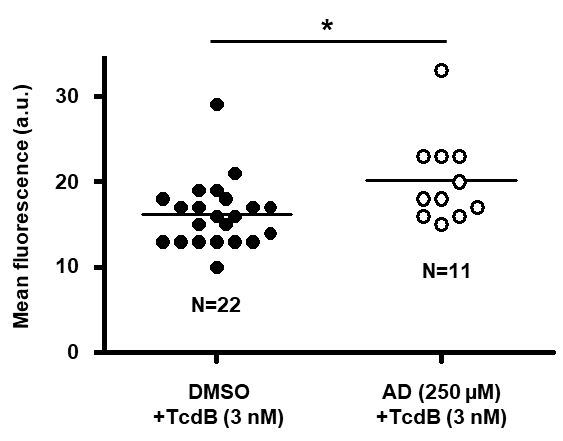


**Supplementary Figure 3.** Effect of 24 h amiodarone preincubation of human ‘miniguts’ on Rac1 modification by TcdB. Experiment was performed and analyzed essentially as described in Figure 8B, but with ‘miniguts’ preincubated for 24 h with 250 µM amiodarone (white filled dots) or DMSO (black filled dots) and intoxication for 3 h with 3 nM TcdB. Asterisk indicates statistical significance between two groups with *p < 0.05.


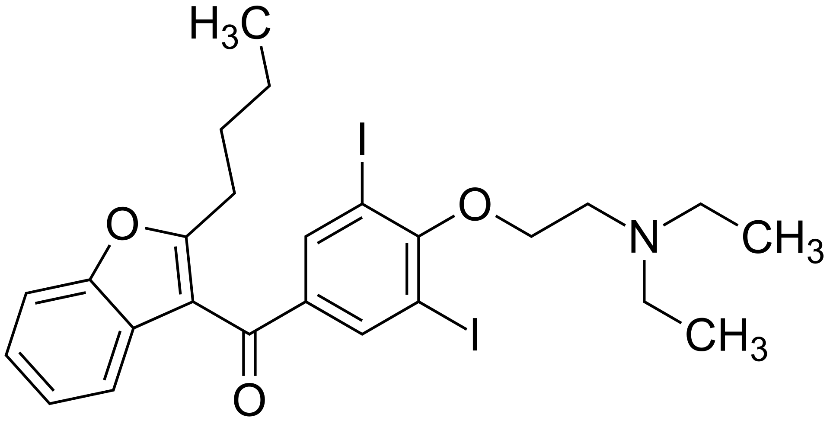


**Supplementary Figure 4.** Chemical structure of amiodarone. IUPAC name: (2-{4-[(2-butyl-1-benzofuran-3-yl)carbonyl]-2,6-diiodophenoxy}ethyl)diethylamine
